# The Application of an Ultra-Thin, High-Density μECoG Array in Dissecting Caffeine-Induced Cortical Dynamics in Mice

**DOI:** 10.3390/s25247552

**Published:** 2025-12-12

**Authors:** Yongqi Hu, Bingjie Zhang, Zhengwei Hu, Xuemei Liu, Xiaojian Li, Ji Dai

**Affiliations:** 1 Shenzhen-Hong Kong Institute of Brain Science, Shenzhen Institute of Advanced Technology, Chinese Academy of Sciences, Shenzhen 518055, China; 2 Guangdong Provincial Key Laboratory of Brain Connectome and Behavior, Shenzhen Institute of Advanced Technology, Chinese Academy of Sciences, Shenzhen 518055, China; 3 University of Chinese Academy of Sciences, Beijing 100049, China; 4 Shenzhen We-Linking Medical Technology Co., Ltd., Shenzhen 518055, China

**Keywords:** chronic caffeine intake, μECoG, sleep spindle, power spectral density, frequency-band coherence

## Abstract

**Highlights:**

**What are the main findings?**

- An ultra-thin 64-channel μECoG array was manufactured for long-term recording.
- Prolonged caffeine intake significantly reduced broadband power spectral density and spindle power.

**What are the implications of the main findings?**

- These findings validate the μECoG array’s functionality.
- Post-chronic caffeine withdrawal lowers cortical oscillatory power yet enhances network connectivity.

**Abstract:**

High-density micro-electrocorticography (μECoG) arrays offer precise spatial resolution with minimal invasiveness. This study employed a custom ultra-thin 64-channel μECoG array to investigate cortical activity in mice under chronic caffeine exposure. While caffeine is known to enhance short-term alertness, its long-term impact on sleep microarchitecture and brain connectivity is unclear. Continuous recordings from adult mice during baseline and recovery revealed that prolonged caffeine intake significantly reduced broadband power spectral density (PSD) and spindle power but increased interregional coherence and altered spindle duration and density. In contrast, six hours of sleep deprivation elevated PSD and coherence, mainly affecting sensorimotor and retrosplenial cortices. These findings validate the μECoG array’s functionality and demonstrate that post-chronic caffeine withdrawal lowers cortical oscillatory power yet enhances network connectivity, whereas acute sleep loss boosts global synchrony. This work clarifies how sustained caffeine use and sleep deprivation distinctly disrupt sleep homeostasis through different neural mechanisms.

## 1. Introduction

Electrocorticography (ECoG) offers a powerful approach by capturing neural activity directly from the cortical surface via electrodes placed beneath the skull but above or below the dura mater, without penetrating brain tissue [1,2]. Compared to conventional scalp electroencephalography (EEG), ECoG provides superior spatial resolution—on the order of millimeters rather than centimeters—along with enhanced signal amplitude, reduced susceptibility to noise, and greater stability for long-term monitoring. These advantages enable more precise mapping of cortical dynamics and better characterization of neural oscillations.

Recent technological advancements have further refined this methodology through the development of micro-electrocorticography (μECoG) arrays [3]. These devices feature high-density electrode configurations that achieve spatial and temporal resolutions approaching those of intracortical microelectrodes, yet with significantly reduced invasiveness. The smaller craniotomy required for μECoG implantation and the minimized volume of implanted material reduce surgical risks and improve biocompatibility, making μECoG a promising tool for both research and clinical applications, including brain–computer interfaces [4,5,6].

In response to the growing need for sophisticated μECoG technology within both fundamental and clinical neuroscience research, we collaborated closely with the engineering team of a biotechnology company to develop an ultra-thin, high-density μECoG array. The proprietary manufacturing techniques for these arrays have been submitted for patent protection in China and are presently under confidentiality. To validate the performance and dependability of this technology in neuroscience applications, we carried out an experiment aimed at characterizing neural activity following extended caffeine exposure in mice. The following sections provide a detailed overview of the study’s background and underscore the critical importance of employing advanced μECoG technology.

Sleep is a conserved, essential biological process required for physiological homeostasis, and its disruption produces widespread adverse effects [7]. Chronic sleep loss, in particular, is associated with cognitive deficits, metabolic and immune dysfunction, and in extreme cases psychosis or death [8]. Despite this, insufficient sleep remains common in modern societies, driven in part by lifestyle factors such as widespread caffeine use, which markedly alters sleep–wake regulation and circadian timing.

Caffeine, a naturally occurring alkaloid found in coffee, tea, chocolate, and numerous energy drinks, stands as the most widely consumed psychoactive substance worldwide [9]. Its popularity stems from its ability to enhance alertness, reduce perceived fatigue, and improve cognitive performance, making it a staple in daily routines across diverse populations. The pharmacological action of caffeine primarily involves its role as a non-selective antagonist of adenosine receptors, particularly A1 and A2A subtypes [10,11,12]. Adenosine is a neuromodulator that accumulates during prolonged wakefulness and promotes sleepiness by inhibiting neural activity. By blocking adenosine receptors, caffeine effectively counteracts this sleep pressure, thereby promoting wakefulness and delaying the onset of sleep [13,14].

Extensive research has documented the acute effects of caffeine on sleep architecture. Short-term administration typically results in decreased total sleep time, increased sleep latency, and fragmented sleep patterns, reflecting its wake-promoting properties [15,16,17]. These findings align with the common experience of caffeine-induced insomnia or difficulty initiating sleep when consumed close to bedtime. However, the impact of post-chronic caffeine withdrawal on sleep dynamics is more complex and less well understood. Emerging evidence from animal studies, particularly murine models, suggests that prolonged caffeine intake may paradoxically enhance certain aspects of sleep. For instance, Panagiotou and colleagues [17] reported that chronic caffeine exposure increased sleep duration and reduced wakefulness during the light phase, indicating potential adaptive or compensatory mechanisms within the central nervous system. This counterintuitive phenomenon raises important questions about the long-term neurophysiological consequences of habitual caffeine use and its influence on the intricate balance between sleep and wake states.

Despite these intriguing observations, the detailed effects of sustained caffeine consumption on the microstructure of sleep and the functional connectivity of neural networks remain inadequately characterized. Existing studies mostly focus on overall spectral power (such as SWA) or macro-level sleep amounts (time proportions), whereas systematic investigations of spectral dynamics and microstructural features—such as spindle duration and density—remain limited. Sleep is not a uniform state but comprises multiple stages, each with distinct electrophysiological signatures and functional roles. Among these, sleep spindles—brief bursts of oscillatory brain activity occurring during non-rapid eye movement (NREM) sleep—are of particular interest due to their involvement in memory consolidation, synaptic plasticity, and cortical development [18,19,20,21,22,23,24]. Understanding how chronic caffeine intake modulates such fine-grained aspects of sleep architecture is crucial for elucidating its broader impact on brain function and health.

To investigate these complex interactions, advanced neurophysiological recording techniques are indispensable. In the present study, we employed 64-channel μECoG arrays to conduct an in-depth examination of post-chronic caffeine withdrawal on sleep physiology in mice. Our comprehensive analysis encompassed multiple dimensions of sleep, including total sleep duration, power spectral density (PSD) across frequency bands, interhemispheric coherence reflecting functional connectivity, and detailed characterization of sleep spindles through parameters such as amplitude, duration, power, and density. In addition to demonstrating the reliability of the μECoG array we co-developed, we aimed to elucidate the nuanced ways in which sustained caffeine intake reshapes sleep architecture and neural network dynamics, thereby advancing our understanding of its impact on brain health and function.

## 2. Materials and Methods

### 2.1. μECoG Array

**Figure 1:**
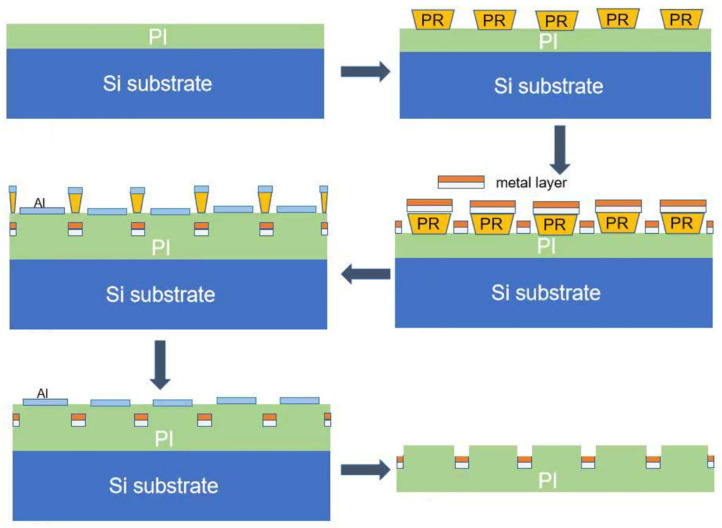
The fabrication process of the μECoG array. PI: Polyimide; Si: Silicon; PR: Photoresist; Al: Aluminum. The metal layer includes a 10 nm-thick titanium adhesion layer and a 100 nm-thick gold layer.

**Figure 2:**
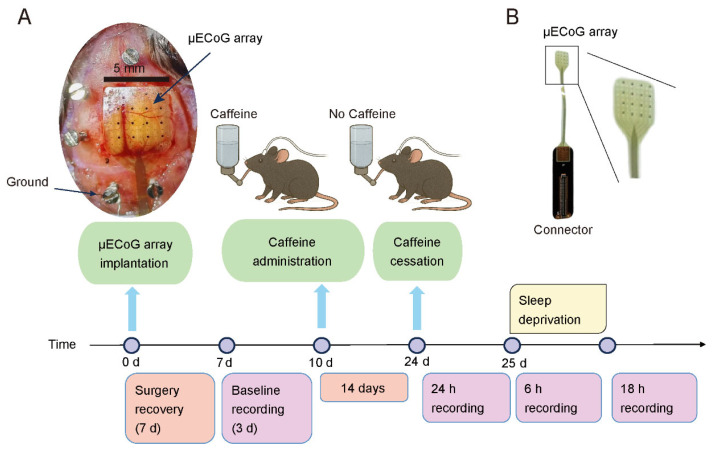
Overview of the experimental procedure for mouse recordings. (**A**) Timeline illustrating the recording process, with the sequence of experimental steps displayed above the time axis and their respective durations indicated below. The image on the top-left shows the location of the implantation and how the array looks like after being implanted. (**B**) The image of the μECoG array with a connector.

The μECoG array utilized in this study was developed by the Shenzhen Institute of Advanced Technology–We Linking Medical Joint Laboratory. The fabrication procedure of the array is illustrated in Figure 1. The process begins with a silicon (Si) wafer serving as the rigid support substrate. A 5 μm-thick polyimide (PI) polymer layer was spin-coated onto the Si wafer and cured at 350 °C to form a flexible support layer for the electrode. This PI layer acts as the structural substrate, providing mechanical flexibility and electrical insulation, while the subsequent metal layers are deposited on its surface to form the conductive pathways. Following this, an AZ 5214E photoresist layer is spin-coated onto the polymer surface, after which the microelectrodes and metal traces are defined through standard photolithography techniques. Upon completion of hard baking, the sample undergoes oxygen plasma treatment to roughen its surface, thereby improving the adhesion between the polymer substrate and the metal layers. Subsequently, the metal layers, including a 10 nm-thick titanium (Ti) adhesion layer and a 100 nm-thick gold (Au) layer, are deposited onto the sample via electron-beam evaporation. The sample is then immersed in acetone to lift off unwanted metal regions, resulting in the formation of the desired metal pattern. A final 5 μm-thick polymer layer is applied on top. The sample is then subjected to oxygen reactive ion etching, using an aluminum (Al) hard mask to expose the electrode sites and bond pads. After the removal of the Al mask, the electrode array is released from the wafer. The distinctive manufacturing techniques utilized in producing these arrays have been secured under Chinese patent protection (Patent numbers: CN118592963B, CN117766198B). In this study, the array employed for mice is a 64-channel model (uCortex0–3), measuring 5 mm by 5 mm with a thickness of 10 μm and an inter-electrode spacing of 600 μm, as shown in Figure 2. The fabrication processes employed in this scheme are all standard micro/nano-fabrication techniques, which are highly transferable and reproducible, facilitating replication on other platforms.

### 2.2. Animals

Three male C57BL/6 mice, approximately 20 weeks old and weighing between 26 and 33 g, were utilized for this study. Two mice served as the experimental group, and the other one served as the control group. The animals were maintained under strictly controlled conditions, including a temperature range of 22–23 °C and a 12-h light/dark cycle with lights on at 7:00 AM. Food and water were provided ad libitum. Prior to data acquisition, the mice were acclimated to the recording environment for a period of three days.

### 2.3. Experimental Procedures

The experimental procedures are illustrated in Figure 2A. Specifically, day 0 was defined as the day of electrode implantation. Following a 7-day postsurgical recovery, baseline recordings were performed starting on Day 7 and continued for 72 h (3 × 24 h). On Day 10, caffeine administration was initiated. Caffeine (CAS: 51168-26-4; Bellingham, Beijing, China) was delivered orally through drinking water at a concentration of 0.8 mg/mL over a two-week period. This concentration significantly affects circadian rhythms [25,26] and is equivalent to the caffeine concentration in ordinary drip coffee [17]. Caffeine water was provided ad libitum for the Caffeine group. Control animals received unadulterated water. After two weeks of treatment, the drug was withdrawn and replaced with regular drinking water. Continuous recordings were resumed on Day 24 for 24 h. On Day 25, the animals underwent 6 h of sleep deprivation during which recordings were obtained, followed by an additional 18 h of recovery recording. The body weight of each animal was regularly monitored throughout the experimental period (Supplementary Figure S1).

### 2.4. Surgical Procedures

All surgical interventions were conducted under gaseous anesthesia. Mice were secured in a stereotaxic frame using ear bars. The scalp and adjacent tissues were carefully excised with scissors, and the skull surface was cleansed with hydrogen peroxide to remove residual tissue, followed by saline rinsing and drying with sterile cotton. Craniotomy sites were determined stereotaxically based on electrode dimensions, typically at coordinates AP: −3 mm and ML: 2 mm, with adjustments tailored to individual brain size. Three to four stainless steel skull screws (0.8 mm diameter, 2 mm length) were implanted, with the ground wire affixed to a screw positioned above the cerebellum. A small craniotomy was performed using a hand drill, and the dura mater was delicately removed using a needle tip. The flexible 64-channel polyimide electrode array was placed directly onto the cortical surface and secured with a biocompatible adhesive, followed by stabilization with dental cement. After a seven-day recovery period, experimental recordings commenced. During sessions, the head-mounted connector was linked to a headstage connected via cable to the acquisition system, with data transmitted to a recording computer through USB.

### 2.5. Recording Procedure

Recordings were obtained via an Intan Technologies preamplifier system (RHD2164, Intan Technologies LLC., Los Angeles, CA, USA), incorporating an online 50 Hz notch filter. Simultaneous video monitoring was conducted using an infrared camera controlled by VideoCap software (version 1.27.0.22, Microsoft Corporation).

Baseline electrophysiological data were recorded continuously for three consecutive days (24 h per day) before any drug intervention, during which the mice received no pharmacological treatment. Subsequently, caffeine was administered orally via drinking water at a concentration of 0.8 mg/mL for two weeks; no recordings were conducted during this administration phase. Following the cessation of caffeine treatment, the animals were returned to plain water, and data collection resumed immediately. Continuous recordings were performed for 24 h on the first day post-withdrawal to capture the acute effects of drug discontinuation. On the following day, mice underwent six hours of sleep deprivation, after which recordings continued uninterrupted for an additional 18 h to evaluate the impact of sleep loss and recovery.

### 2.6. Electrophysiological Data Analysis

Sleep–wake states were classified in 4-s epochs as wakefulness, non-rapid eye movement (NREM) sleep, or rapid eye movement (REM) sleep, integrating μECoG signals and video data. Epochs exhibiting body movement in video frames were scored as wake; otherwise, classification relied on dominant frequency power derived from short-time Fourier transform (STFT) analyses. Sleep staging adhered to a simple version of widely-accepted mouse scoring guidelines [27,28], employing frequency bands of 8–30 Hz dominant for wake, 0.5–4 Hz dominant for NREM, and 6.25–9 Hz dominant for REM. Video processing involved extracting one frame every 4 s and detecting motion within a predefined region of interest using frame differencing, thresholding, and morphological operations. Data analysis and sleep scoring were performed blinded to treatment/condition.

Power spectral density (PSD) of μECoG signals was computed over 0.5–150 Hz during the same time period of the light phase daily (PSD computed from a 15 s segment from ZT1.5–2 each day for caffeine group, ZT0–0.16 for control mouse). Notch filters at 50, 100, and 150 Hz were applied to mitigate noise. PSD estimation utilized the Welch method with 2-s windows and 1.75-s overlap. The 8 × 8 matrix of spatial PSD distribution was interpolated via a radial basis function (RBF) kernel to 100 × 100 matrix. Number of contours was 20.

Coherence analysis quantified synchronization between electrode channels. Coherence $C_{xy}(f)$ was defined as:

(1) $$C_{xy}(f)=\frac{|P_{xy}(f){|}^{2}}{P_{xx}(f)P_{yy}(f)}$$

where $P_{xy}(f)$ denotes the cross-power spectral density between signals x(t) and y(t), and $P_{xx}(f)$ and $P_{yy}(f)$ represent their respective power spectral densities. Coherence values range from 0 (no correlation) to 1 (perfect correlation). Calculations were performed across six frequency bands—delta (1–4 Hz), theta (4–8 Hz), alpha (8–12 Hz), beta (12–30 Hz), gamma1 (30–80 Hz), and gamma2 (80–150 Hz)—using 15-s data segments collected at the same daily time points (e.g., starting at 7:00 or 19:00) before and after drug administration (Control mouse at the same time period). Data were arranged according to the electrode layout, bandpass filtered (1–150 Hz), and notch filtered at 50, 100, and 150 Hz. The Chronux toolbox (http://chronux.org/, accessed on 11 November 2025) facilitated coherence computation. Statistical differences in coherence pre- and post-drug administration were assessed via the Wilcoxon matched-pairs signed-rank test. To account for multiple comparisons, the false discovery rate (FDR) correction was applied. Significance thresholds are indicated as * (*p* < 0.05), ** (*p* < 0.01), and *** (*p* < 0.001). All statistical analyses were conducted within individual animals.

Spindle detection employed a continuous region segmentation combined with statistical filtering. Raw signals were downsampled to 1024 Hz; spikes exceeding five standard deviations were excised and replaced by linear interpolation. A second-order Butterworth high-pass filter at 0.5 Hz detrended the data, followed by independent component analysis (ICA) with 32 components. Subsequently, a fifth-order Butterworth bandpass filter (10–16 Hz) isolated spindle activity. Spindle events were identified as signals with envelope amplitudes exceeding the mean plus/minus 1.5 standard deviations of NREM baseline, lasting between 0.4 and 3 s within the NREM stage [29]. Spindle amplitude is defined as the difference between the maximum and minimum amplitudes observed during a spindle event (Supplementary Figure S2). Spindle power refers to the power spectral density (PSD) value calculated for a single spindle. The duration of a spindle corresponds to the length of time, measured in seconds, that an individual sleep spindle persists. Lastly, spindle density is quantified as the frequency of spindles occurring per unit of time, expressed in units of s^−1^.

(2) $$Spindle\;density=\frac{number\;of\;spindles}{duration}$$

For each spindle metric (amplitude, duration, power, density) we fitted a linear mixed-effects model to test the effect of time (Before vs. After). Models were implemented in Python 3.12 using the “statsmodels”. The dependent variable was the metric of interest and the model included time as a fixed effect and mouse_id as a grouping factor (random intercept) to account for within-animal clustering: metric ~ time + (1 | mouse_id). Models were fitted using restricted maximum likelihood (REML) for parameter estimation. Fixed-effect estimates are reported as β ± SE, with 95% confidence intervals and associated test statistics. 

## 3. Results

### 3.1. Post-Chronic Caffeine Withdrawal Prolongs Sleep Duration and Diminishes Wakefulness

**Figure 3:**
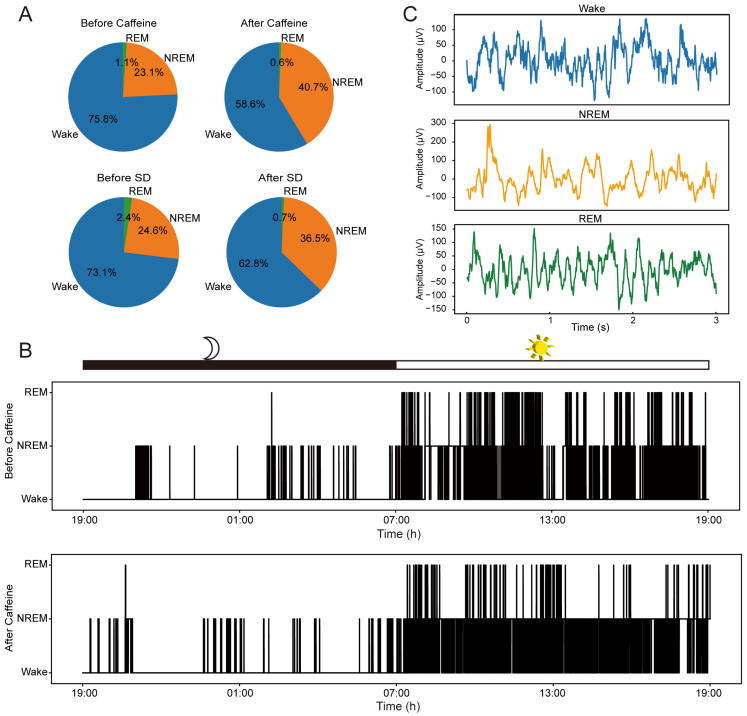
Behavioral impacts on sleep patterns and exemplar μECoG recordings. (**A**) Distribution percentages of Wakefulness, NREM, and REM stages observed prior to and following caffeine administration (n = 2) and sleep deprivation (n = 1). (**B**) Sleep architecture of one representative animal throughout an entire light–dark cycle, comparing conditions before and after prolonged caffeine consumption. (**C**) Representative μECoG traces from one representative animal corresponding to the three distinct sleep stages.

Firstly, we report the general effect of caffeine on sleep behavior in mice. Our investigation revealed that sustained caffeine intake significantly extended total NREM sleep time while concurrently reducing periods of wakefulness. By classifying sleep stages, we quantified the relative time spent in each stage over an approximately 24-h cycle (Figure 3). As illustrated in Figure 3A, the proportion of NREM sleep increased in the chronic caffeine group, rising from 23.1 ± 1.3% (mean ± SD, n = 2) pre-intake to 40.7 ± 9.1% post-intake, whereas wakefulness decreased from 75.8 ± 0% to 58.6 ± 8.5% (comprehensive data are provided in Supplementary Table S1). This shift was similar to the effects observed following short-term (6-h) sleep deprivation, where NREM sleep increased from 24.6% to 36.5% (n = 1) and wakefulness declined from 73.1% to 62.8%. Figure 3B further depicts sleep–wake patterns across a full circadian cycle before and after caffeine exposure, confirming heightened activity during the dark phase and increased sleep during the light phase. 

Figure 3C displays representative ECoG signals corresponding to the three sleep stages, characterized by clear, noise-free waveforms and distinct patterns unique to each sleep cycle. These observations are consistent with prior research [17], providing a reliable foundation for subsequent analyses aimed at elucidating the impact of caffeine exposure on neural dynamics.

### 3.2. Attenuation of Cortical Activation Following Prolonged Caffeine Exposure

**Figure 4:**
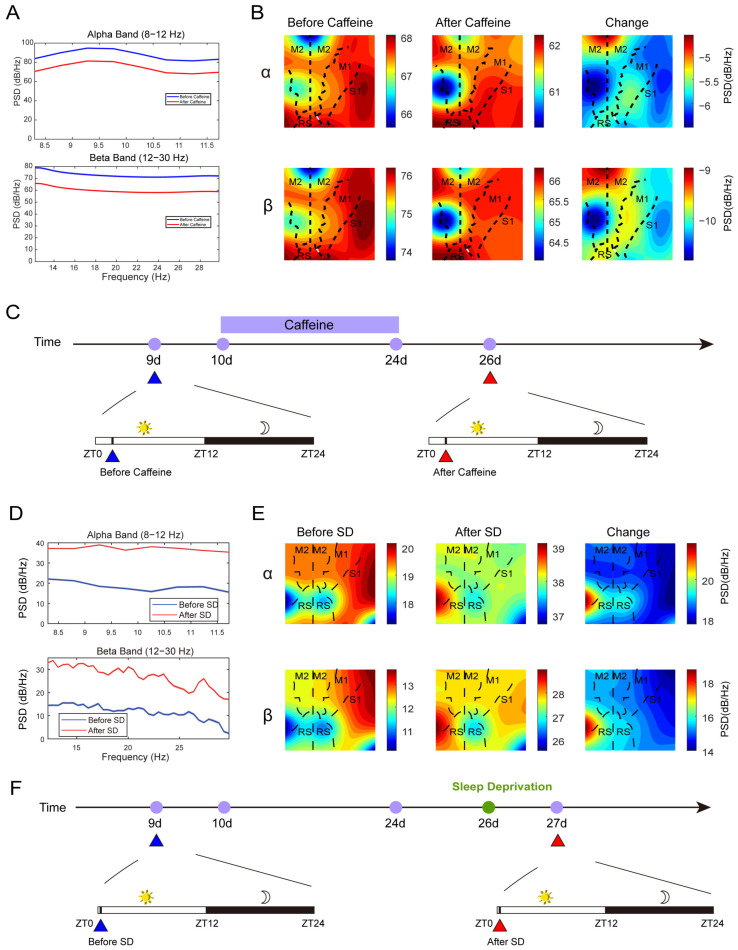
The outcomes of the Power Spectral Density (PSD) analysis. (**A**,**B**): The PSD values of the alpha and beta bands, along with their corresponding spatial distributions, measured before and after prolonged caffeine consumption. (**A**) is one mouse’s multi-channel average data. Data taken from ZT1.5–2. (**B**) is 10log10 of PSD. (**C**,**F**): The corresponding timeline. (**D**,**E**): The PSD of the alpha and beta bands and their spatial patterns prior to and following sleep deprivation (SD: ZT6–12). Data taken from ZT0–0.16. In panels (**B**,**E**), the vertical dashed line demarcates the division between the left and right cerebral hemispheres. The abbreviations used are as follows: M1 denotes the primary motor cortex; M2, the secondary motor cortex; S1, the primary somatosensory cortex; and RS, the retrosplenial cortex. SD refers to sleep deprivation.

Next, we focus on reporting the neural dynamics revealed by the μECoG array. Power spectral density (PSD) analysis of μECoG recordings obtained during the light phase (a 15 s segment from ZT1.5–2 each day) demonstrated a reduction in alpha (8–12 Hz) and beta (12–30 Hz) band activity within the caffeine group (Figure 4A, see Supplementary Figure S3 for raw sample data, Figures S4–S6 for each animal and different phases, Figure S7 for longer time period), indicative of diminished cortical activation. In contrast, the 6-h sleep deprivation (ZT6–12) cohort exhibited elevated PSD values (a 15 s segment from ZT0–0.16 each day), reflecting enhanced cortical excitability (Figure 4B). Spatial mapping of PSD changes revealed that although reductions were widespread, they were most prominent in the S1 region of the right hemisphere (Figure 4C, see Supplementary Figure S8 for the channel layout mapping). Conversely, sleep-deprived mice showed increased PSD, particularly in the S1 area of the left hemisphere (Figure 4D), highlighting distinct regional modulation of cortical activity under these conditions.

### 3.3. Augmented Functional Connectivity Between Brain Regions

**Figure 5:**
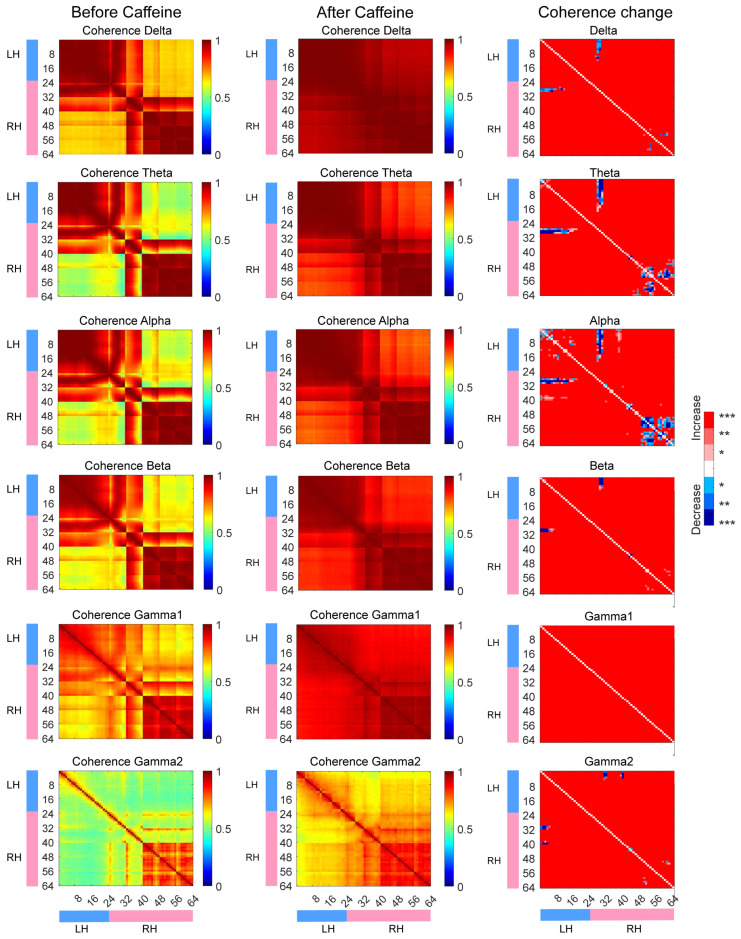
The coherence measurements obtained prior to and following caffeine administration, alongside the respective alterations observed. LH refers to the left hemisphere, while RH denotes the right hemisphere. In the figure, red signifies an increase in coherence, blue represents a decrease, and white indicates no significant change. Statistical significance is marked as follows: * *p* < 0.05, ** *p* < 0.01, and *** *p* < 0.001.

Further, frequency-domain coherence analysis of μECoG signals was employed to assess interregional functional coupling. As shown in Figure 5, coherence across the 1–150 Hz frequency range—including delta (1–4 Hz), theta (4–8 Hz), alpha (8–12 Hz), beta (12–30 Hz), gamma1 (30–80 Hz), and gamma2 (80–150 Hz) bands—was significantly elevated in the caffeine group across most channels (*p* < 0.001). This enhancement mirrored patterns observed in the sleep deprivation group (Supplementary Figure S9), with increased coherence both within and between hemispheres. In contrast, the control group showed reduced coherence across most brain regions (Supplementary Figure S10). These findings demonstrate that post-chronic caffeine withdrawal modulates neural synchrony by strengthening functional connectivity. Notably, coherence within the theta and alpha bands between specific channels—namely M2 and RS regions in the right hemisphere and M2, M1, and RS regions in the left hemisphere—was either diminished or unchanged.

### 3.4. Modifications in Spindle-Related Characteristics

**Figure 6:**
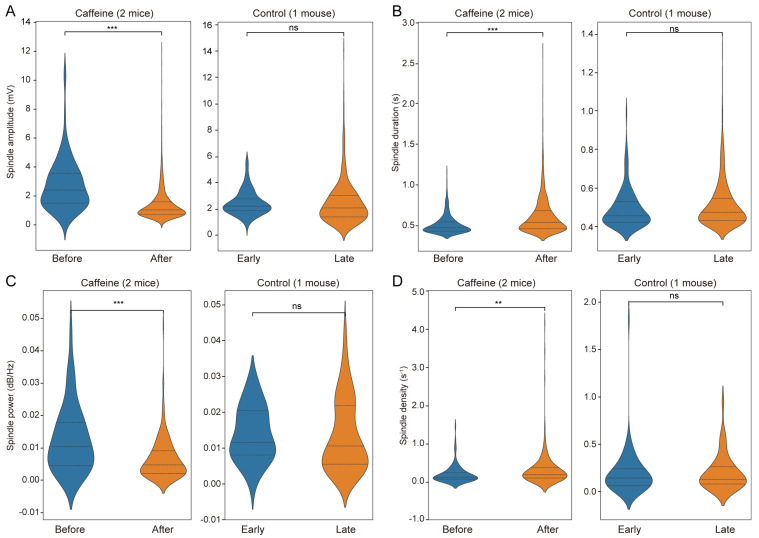
Analysis of spindle characteristics in caffeine-treated and control groups. (**A**) Spindle amplitude (Caffeine: n = 1963 observations, β = 1.39 ± 0.11 SE, z = 12.297, 95% CI [1.17, 1.61], *p* < 0.001; Control: n = 618 observations), (**B**) spindle duration (Caffeine: n = 2413 observations, β = −0.078 ± 0.015 SE, 95% CI [−0.108, −0.049], *p* < 0.001; Control: n = 619 observations), (**C**) spindle power (Caffeine: n = 236 observations, β = 0.006 ± 0.001 SE, z = 5.219, 95% CI [0.004, 0.008], *p* < 0.001; Control: n = 159 observations), and (**D**) spindle density (Caffeine: n = 374 observations, β = −0.109 ± 0.042 SE, z = −2.601, 95% CI [−0.192, −0.027], *p* < 0.01; Control: n = 124 observations). The left side displays the variations observed pre- and post-caffeine administration, while the right side presents the equivalent measurements for the control group. Control refers to a separate animal. ** *p* < 0.01, *** *p* < 0.001, and ns: not significant.

Spindles are ubiquitous oscillations during NREM sleep and commonly serve as a neural signature in sleep studies [30,31]. In the current study, spindle events during NREM sleep were detected before and after treatment across all groups. Key spindle parameters—including amplitude, duration, power, and density—were quantified (Supplementary Figure S2). The caffeine group exhibited a significant reduction in spindle amplitude (n = 1963 observations, β = 1.39 ± 0.11 SE, z = 12.297, 95% CI [1.17, 1.61], *p* < 0.001), with a distribution skewed toward lower values (Figure 6A), whereas no significant changes occurred in controls (n = 618 observations). This suggests that post-chronic caffeine withdraw impairs neuronal synchronization, potentially compromising NREM sleep quality. Conversely, spindle duration increased significantly following caffeine intake (n = 2413 observations, β = −0.078 ± 0.015 SE, 95% CI [−0.108, −0.049], *p* < 0.001) without notable alterations in the control group (Figure 6B), possibly reflecting enhanced stability of neuronal oscillations and deeper NREM sleep or prolonged memory consolidation processes. Spindle power was significantly decreased in caffeine-treated mice (n = 236 observations, β = 0.006 ± 0.001 SE, z = 5.219, 95% CI [0.004, 0.008], *p* < 0.001), with no significant variation in controls (Figure 6C), indicating reduced overall network activity that may adversely affect NREM sleep integrity or cognitive functions.

Spindle density, defined as the number of spindles per second, was also assessed. The caffeine group demonstrated a significant increase in spindle density (n = 374 observations, β = −0.109 ± 0.042 SE, z = −2.601, 95% CI [−0.192, −0.027], *p* < 0.01) accompanied by a broader distribution (Figure 6D). High-density episodes suggest periods of intense spindle activities within short intervals, implying that NREM sleep stages became more frequently associated with spindle occurrences following chronic caffeine exposure. This pattern may reflect a compensatory mechanism after caffeine cessation, resulting in more active spindle events in NREM sleep. No significant differences in spindle density were observed in the control group.

## 4. Discussion

### 4.1. The Manufacturing Advancement of the Lab-Developed μECoG Array

To situate this work within the broader academic landscape, we conducted a comparative analysis against previously reported μECoG arrays of comparable dimensions. Prior research [32,33] has established that gold electrodes with diameters or side lengths near 50 μm generally exhibit impedances in the megaohm range at 1 kHz, highlighting a persistent challenge inherent to microscale electrodes. The initial impedance measurements of the 50 μm gold electrodes fabricated in this study align with these established findings.

Elevated impedance levels pose a significant obstacle by degrading the signal-to-noise ratio (SNR) of neural recordings, primarily through increased thermal noise and signal attenuation caused by the voltage-divider effect within the recording system [34,35,36].

To overcome this limitation, we implemented an innovative yet straightforward electrochemical roughening technique. Our investigations revealed that applying a precisely controlled voltage treatment to the electrode surfaces induces the development of intricate microstructures, markedly enhancing the effective surface area. Given that electrode impedance inversely correlates with interfacial area, this approach effectively reduced impedance from the megaohm scale to approximately 100 kΩ or lower. This targeted surface modification strategy offers a robust solution for optimizing the electrical characteristics of microscale electrodes, thereby facilitating the acquisition of high-fidelity neural signals.

### 4.2. The Functional Completeness of the Lab-Developed μECoG Array

This study rigorously establishes the functional comprehensiveness of the ultra-thin, high-density μECoG array in analyzing caffeine-induced cortical dynamics in mice. The effective deployment of this μECoG array, capable of encompassing extensive cortical regions, offers strong evidence that post-chronic caffeine withdrawal and acute sleep deprivation distinctly and fundamentally alter cortical spectral activity. Specifically, prolonged caffeine withdrawal induces a reduction in broadband PSD, whereas acute sleep deprivation leads to widespread elevations across the PSD spectrum. These findings are consistent with prior research demonstrating that chronic caffeine diminishes slow-wave activity in murine models [17] and that short-term sleep loss enhances low-frequency power during NREM sleep recovery phases [37,38]. Importantly, by employing high-density μECoG recordings alongside advanced spectral decomposition methodologies, our study extends these earlier observations, revealing that the caffeine-induced suppression and deprivation-induced augmentation of PSD are not only observable but also spatially extensive, spanning multiple frequency bands across the cortex.

### 4.3. The Efficacy of the μECoG Array in Revealing Sleep-Related Signatures

Previously, Thölke et al. [39] demonstrated that caffeine inhibits low-frequency oscillations, specifically in the delta and theta bands, while simultaneously enhancing power within the sigma and beta frequency ranges. In a similar vein, Landolt et al. [40,41] found that an acute 200 mg dose of caffeine in humans selectively diminishes EEG power in the 0.25–0.5 Hz and 0.75–2.0 Hz bands during NREM sleep, yet increases power in spindle-associated frequencies (11.25–14.0 Hz and 11.25–20.0 Hz). Contrasting these findings, our investigation revealed a marked reduction in spindle power following prolonged caffeine intake, indicating that chronic caffeine intake may suppress oscillatory activity within the spindle frequency domain. This finding aligns with the notion that caffeine disrupts sleep architecture by modulating specific neural oscillations critical for sleep stability and memory consolidation. Conversely, sleep deprivation not only elevated low-frequency power but also substantially increased interhemispheric coherence. While previous studies, such as Vyazovskiy et al. [42], reported enhanced delta-band interhemispheric coherence after sleep deprivation, our data indicate that this coherence enhancement extends beyond the delta band into higher frequency ranges, implying a more comprehensive reorganization of network synchrony under conditions of heightened sleep pressure. This broader coherence augmentation may reflect compensatory neural mechanisms aimed at maintaining functional connectivity despite the deleterious effects of sleep loss.

Collectively, these results suggest a paradoxical role of post-chronic caffeine withdrawal in modulating sleep pressure and brain function. Although acute caffeine consumption transiently promotes wakefulness by antagonizing adenosine receptors [13,14], it does not prevent the progressive accumulation of sleep debt. Instead, sustained caffeine use may appear to elevate sleep pressure over time, thereby compromising neural processes typically associated with restorative sleep. This is evidenced by the observed reductions in slow-wave and spindle activity [43,44], which are hallmarks of sleep homeostasis. Upon cessation of caffeine intake, compensatory mechanisms emerge, characterized by prolonged sleep duration and diminished wakefulness, reflecting an intrinsic homeostatic drive to restore physiological equilibrium. This dynamic underscores the complexity of caffeine’s impact on sleep regulation, wherein short-term benefits for alertness may be offset by long-term disruptions in sleep architecture and recovery.

Our study advances the field by providing a more comprehensive spatial and temporal characterization of caffeine’s effects on sleep regulation than previous investigations. The use of μECoG arrays enabled high-resolution mapping of cortical oscillatory changes, revealing nuanced alterations across multiple frequency bands and cortical regions.

### 4.4. Limitations

Nonetheless, this study presents several notable limitations. The sample size is limited, comprising only two mice in the caffeine group and a single mouse in the control group, which restricts the generalizability of the findings. Furthermore, the absence of recordings during the caffeine administration phase somewhat undermines the assessment of the effects of prolonged caffeine intake; however, the data can still be interpreted as reflecting post-chronic caffeine withdrawal phenomena. Another constraint is the lack of electromyography (EMG) data for sleep staging, which may marginally reduce the precision of REM and NREM sleep classification. Additionally, our automated μECoG plus video analysis pipeline was not validated against expert manual scoring in an independent cohort. Given the extensive duration of recordings—exceeding dozens of terabytes—comprehensive manual scoring was impractical, rendering a streamlined automated method the only feasible approach within our temporal and computational limitations. It is worth noting that Cusinato et al. [45] utilized accelerometry, which captures mouse movement, alongside electrophysiological recordings for sleep staging, an approach conceptually akin to ours. Similarly, McShane et al. [46] demonstrated that video analysis can effectively assess rapid eye movement (REM) sleep in mice, supporting the utility of video data in sleep staging. Moreover, Rahimi et al. [47] confirmed the viability of conducting sleep staging without EMG. Consistent with established methodologies (e.g., Oishi et al. [28]; Leemburg et al. [27]), we designated epochs as NREM when the μECoG spectral profile was dominated by delta waves (0.5–4 Hz), a frequency band widely recognized as a definitive marker of NREM sleep.

### 4.5. The Implication of the Current Study on Future Directions

Nevertheless, the physiological effects of caffeine are multifaceted and likely extend beyond sleep and neural oscillations. Caffeine’s systemic actions, including its impact on cardiovascular function, metabolism, and neurochemical signaling, warrant further investigation to fully appreciate its broad biological consequences. Moreover, the interplay between psychostimulant-induced wakefulness and the evolutionarily conserved homeostatic mechanisms governing sleep remains an open question. Understanding how these opposing forces balance at the molecular, cellular, and network levels is essential for unraveling the complex regulation of sleep and wakefulness.

Future research should aim to dissect the mechanistic underpinnings of caffeine’s modulation of sleep homeostasis. For instance, exploring the role of adenosine receptor subtypes in mediating caffeine’s effects on specific neural circuits could provide valuable insights. Additionally, longitudinal studies examining the cumulative impact of chronic caffeine intake on sleep architecture and cognitive function are needed to assess potential long-term consequences. Investigations employing multimodal approaches, integrating electrophysiology, neuroimaging, and molecular techniques, will be instrumental in delineating the pathways through which caffeine influences brain function.

Furthermore, it is imperative to consider individual variability in caffeine metabolism and sensitivity, which may modulate its effects on sleep and neural dynamics. Genetic factors, age, and lifestyle variables could all contribute to differential responses [48,49], highlighting the need for personalized approaches in caffeine consumption recommendations. Finally, given the increasing prevalence of psychostimulant use in modern society, understanding how these substances interact with intrinsic sleep regulatory systems will be crucial for informing public health policies and clinical interventions aimed at optimizing sleep health.

## 5. Conclusions

In conclusion, the utilization of the ultra-thin, high-density μECoG array to investigate caffeine-induced neural dynamics reveals the intricate and bidirectional impacts of post-chronic caffeine withdrawal and acute sleep deprivation on cortical spectral patterns and network synchrony. This work substantiates the functional integrity of the laboratory-developed μECoG device. By broadening the spatial and frequency domain analysis of these effects, we establish a critical framework for future research aimed at elucidating the underlying mechanisms through which caffeine modulates sleep regulation.

## 6. Patents

The manufacturing techniques utilized in producing the μECoG arrays have been secured under Chinese patent protection (Patent numbers: CN118592963B, CN117766198B).

## Data Availability

The raw data supporting the conclusions of this article will be made available by the authors on request.

## Supplementary Materials

The following supporting information can be downloaded at: https://www.mdpi.com/article/10.3390/s25247552/s1, Figure S1: Longitudinal body weight of each mouse. Figure S2: One example of the spindle waves. Figure S3: Raw data samples at the 9th day and 38th day post-implantation. Figure S4: PSD analyses of two mice in the caffeine group. Figure S5: PSD analysis of one mouse in the control group. Figure S6: PSD analyses of one mouse in the caffeine group and one mouse in the control group. Figure S7: PSD analysis of two-minute data. Figure S8: Channel layout mapping of the electrode array. Figure S9: The coherence measurements obtained prior to and following sleep deprivation. Figure S10: Coherence in the blank control group. Table S1. The percentage of Wake, NREM, and REM for each mouse in the Caffeine group.

## Abbreviations

The following abbreviations are used in this manuscript:

μECoG	Micro-electrocorticography
PSD	Power spectral density
SD	Sleep deprivation
NREM	Non-rapid eye movement
REM	Rapid eye movement
