# Supplementary material for: The Application of an Ultra-Thin, High-Density μECoG Array in Dissecting Caffeine-Induced Cortical Dynamics in Mice
Source: Sensors (Basel). 2025 Dec 12;25(24):7552. doi: 10.3390/s25247552 (PMC12737276; doi:10.3390/s25247552)
Supplement: Supplementary file 1 [file sensors-25-07552-s001.zip › sensors-3984620-supplementary.pdf]

Supplementary Materials

Supplementary Figures

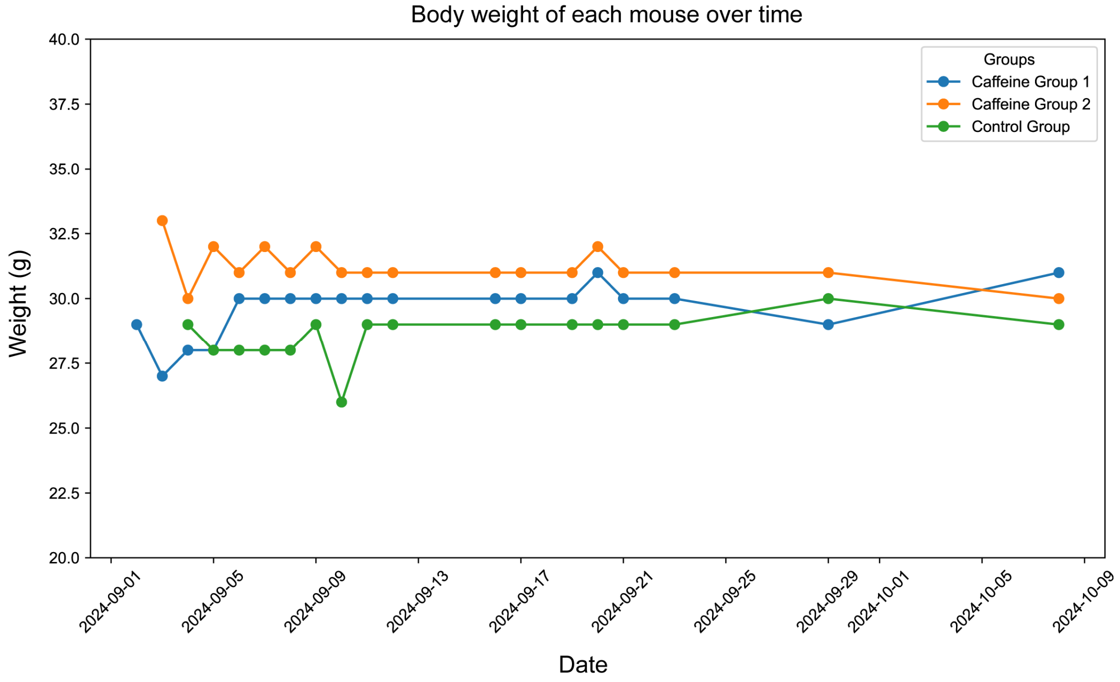

Supplementary Figure S1. Longitudinal body weight of each mouse.

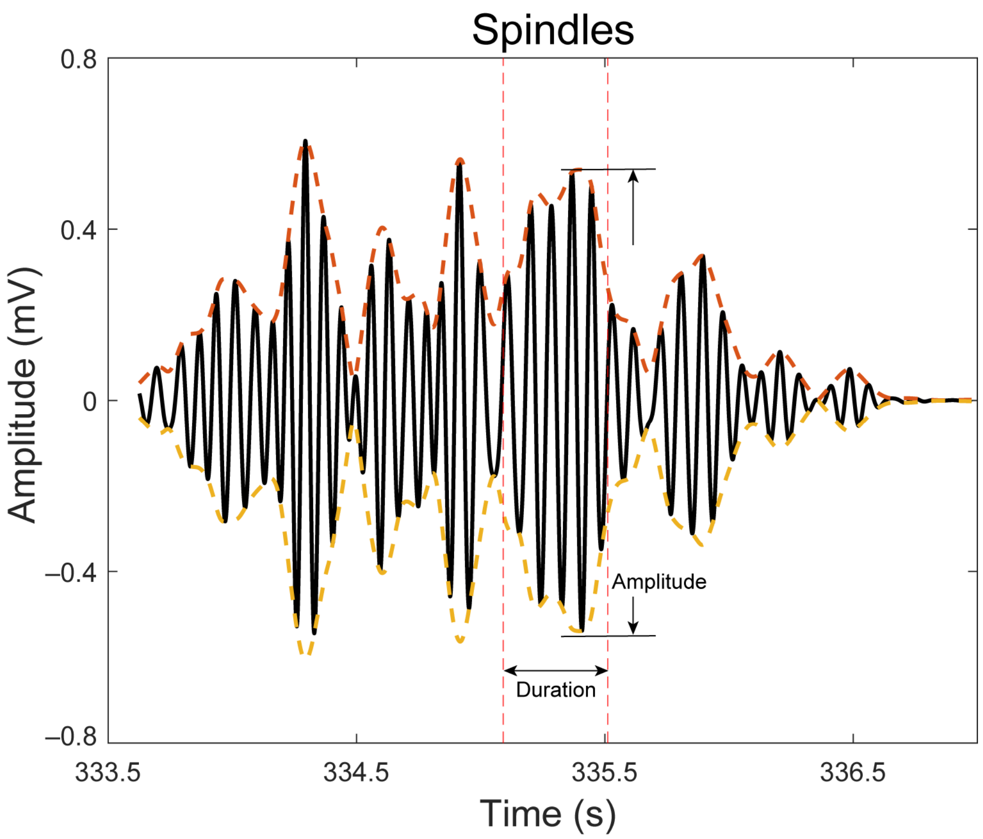

Supplementary Figure S2. One example of the spindle waves. Arrows illustrate the definitions of amplitude and duration.

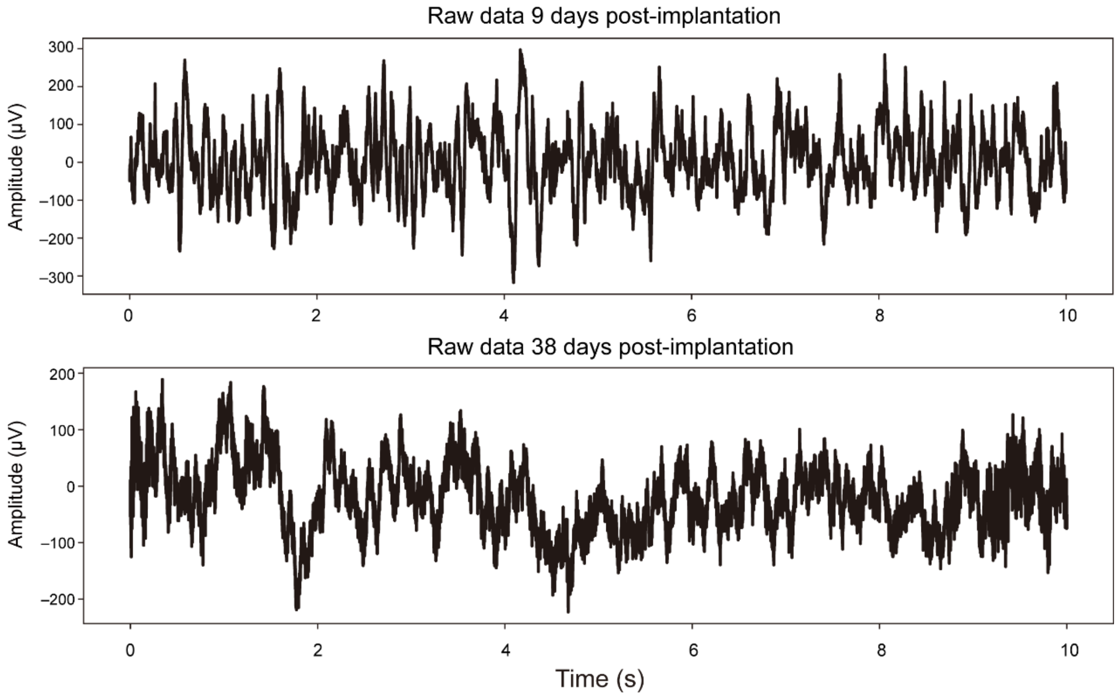

Supplementary Figure S3. Raw data samples at the 9th day and 38th day post-implantation.

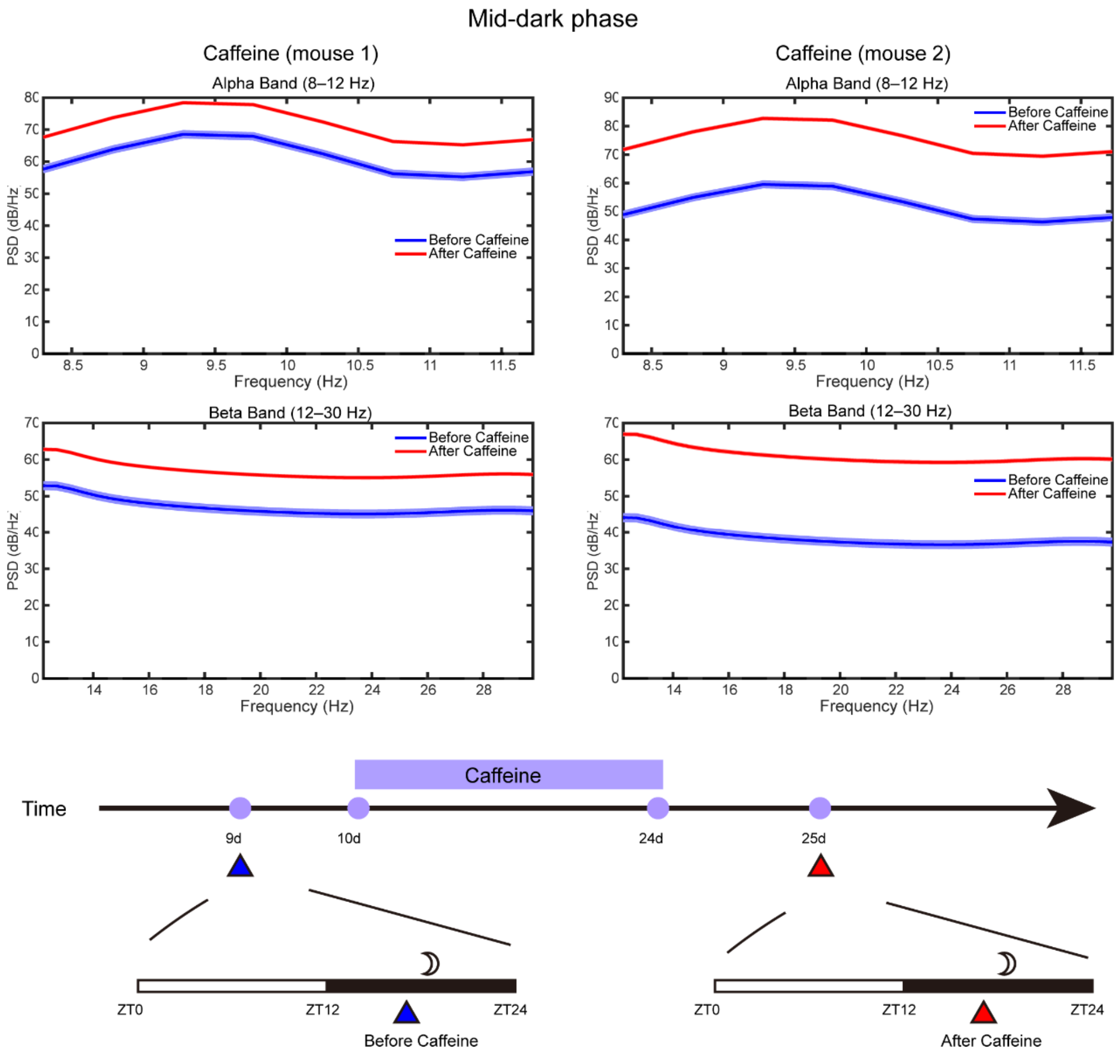

Supplementary Figure S4. PSD analyses of two mice in the caffeine group. The bottom charts the time window for analysis (ZT16.5-17).

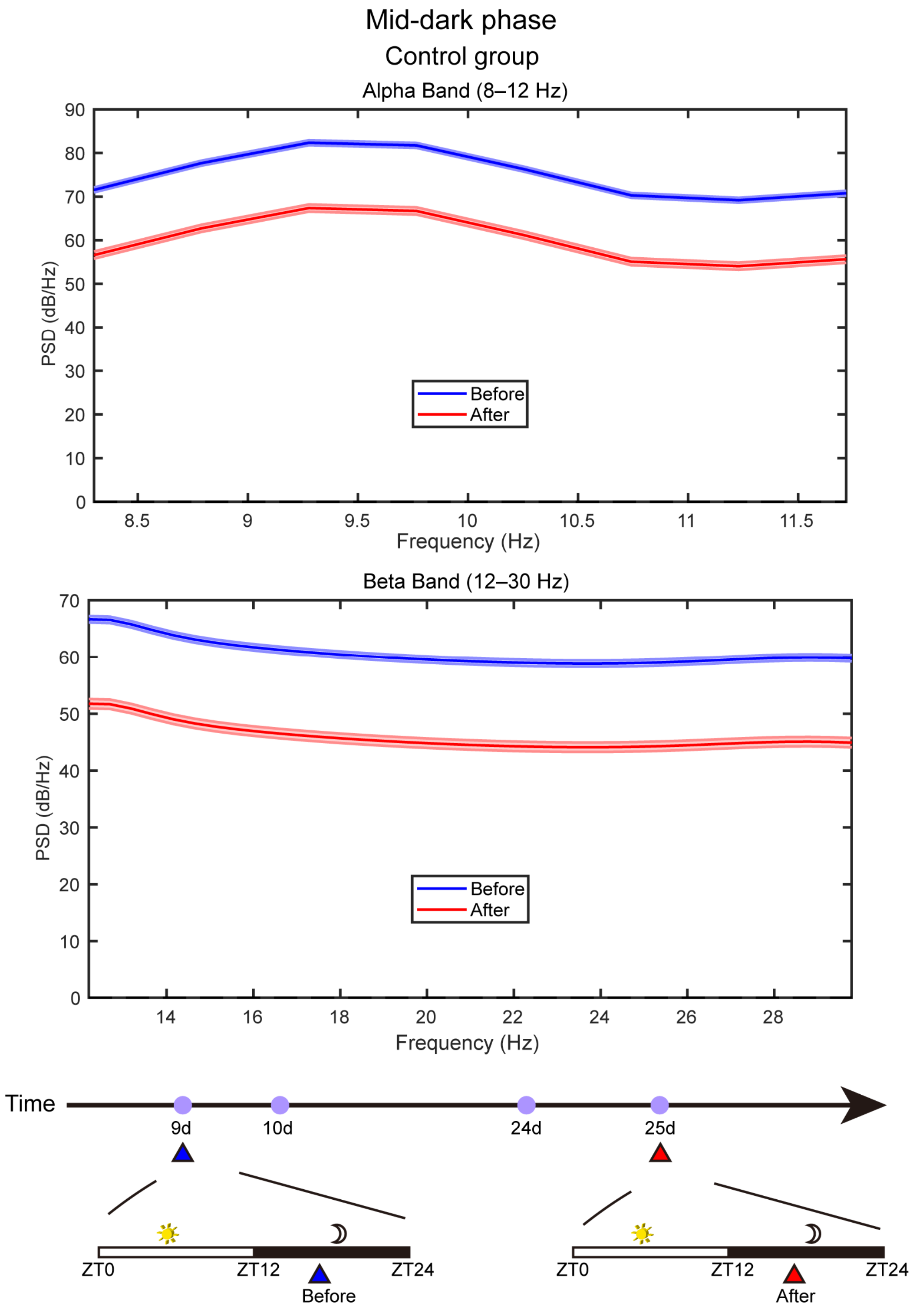

Supplementary Figure S5. PSD analysis of one mouse in the control group. The bottom charts the time window for analysis (ZT16.5-17).

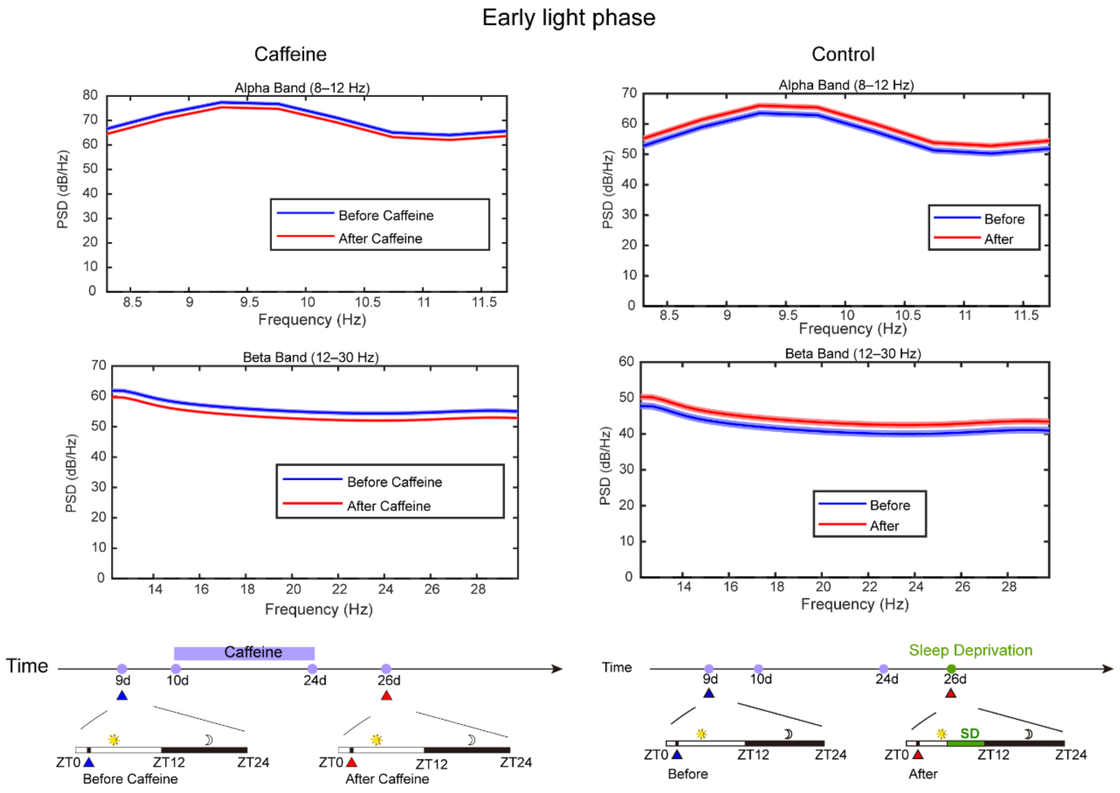

Supplementary Figure S6. PSD analyses of one mouse in the caffeine group and one mouse in the control group. The bottom charts the time window for analysis (ZT0-2). Related to Figure 4A.

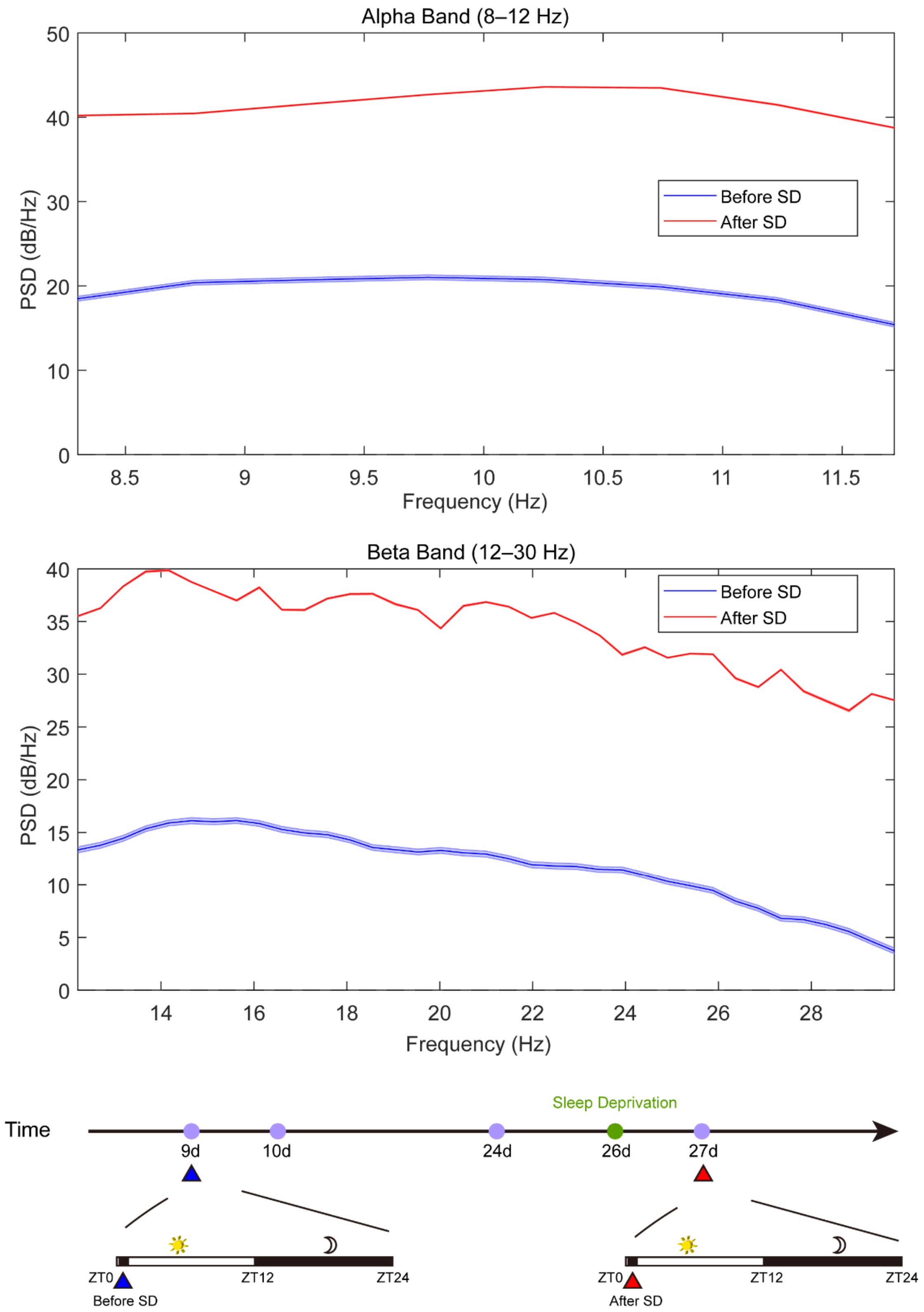

Supplementary Figure S7. PSD analysis of two-minute data. Data samples are the same as in Figure 4D.

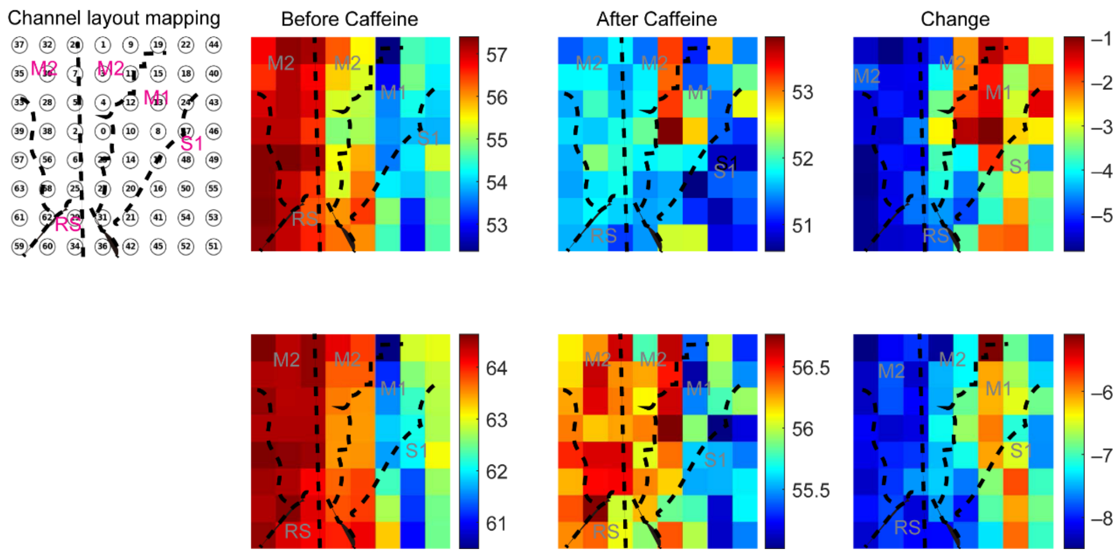

Supplementary Figure S8. Channel layout mapping of the electrode array (8×8 matrix).

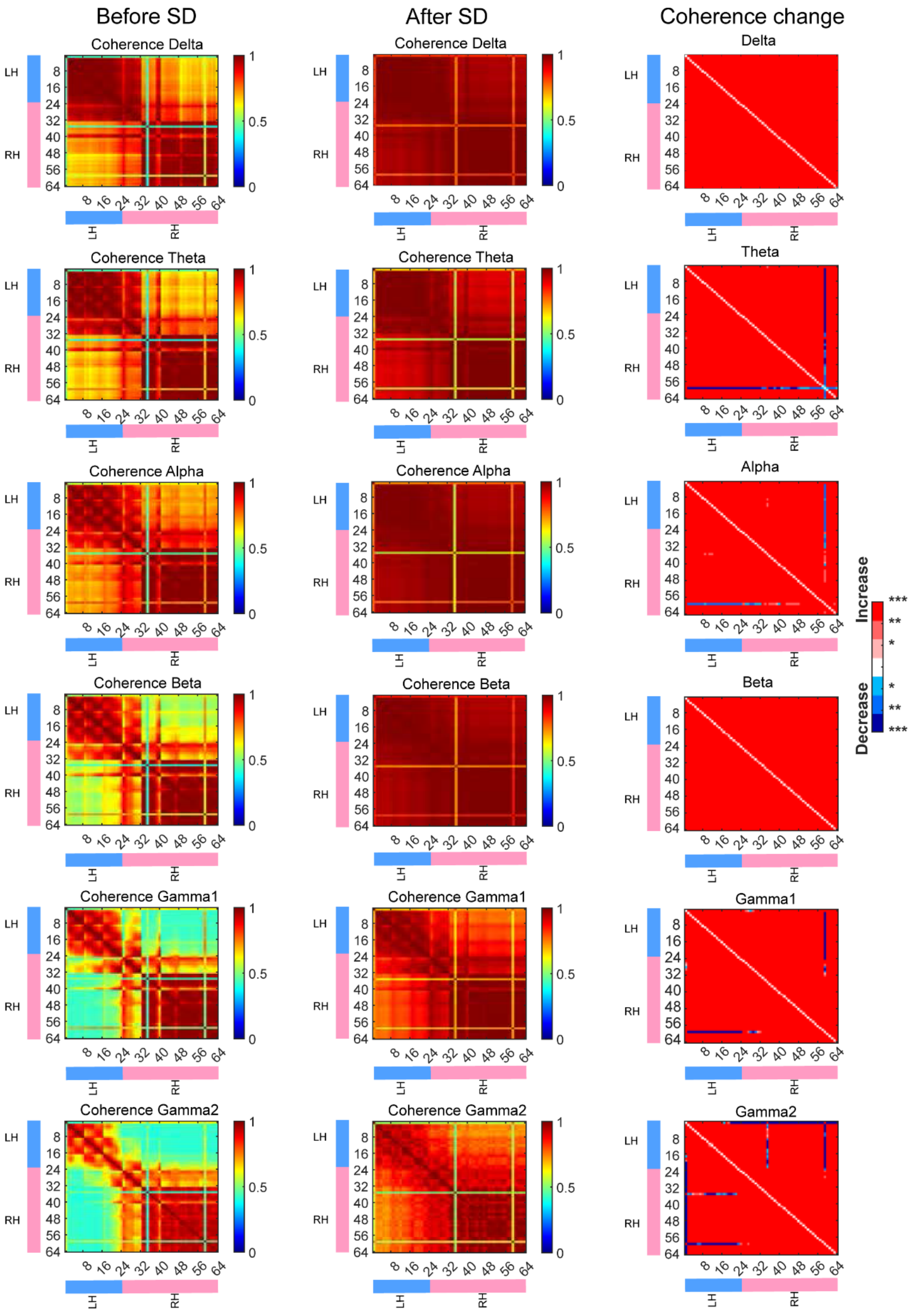

Supplementary Figure S9. The coherence measurements obtained prior to and following sleep deprivation, alongside the respective alterations observed.

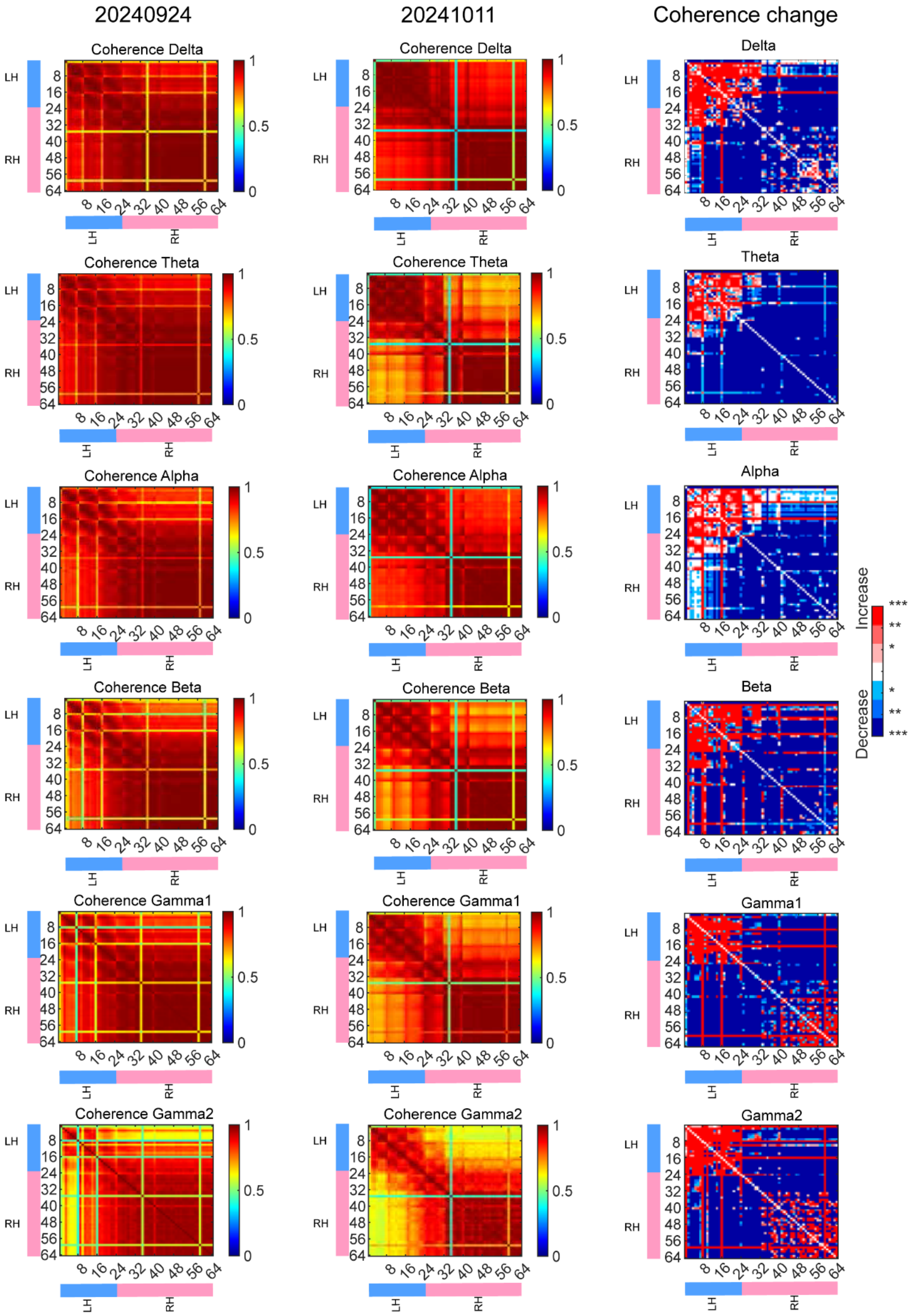

Supplementary Figure S10. Coherence in the blank control group at two time points.

Supplementary Table S1. The percentage of Wake, NREM, and REM for each mouse in the Caffeine group.

	Wake		NREM		REM	
	Before	After	Before	After	Before	After
Mouse1(Caffeine)	75.8%	64.6%	22.1%	34.3%	2.1%	1.1%
Mouse2(Caffeine)	75.8%	52.6%	24.0%	47.2%	0.1%	0.2%
Mean±SD	75.8±0%	58.6±8.5%	23.1±1.3%	40.7±9.1%	1.1±1.4%	0.6±0.6%
